# A life more ordinary: A peer research method qualitative study of the Feeling Safe Programme for persecutory delusions

**DOI:** 10.1111/papt.12421

**Published:** 2022-08-08

**Authors:** Jessica Bond, Alexandra Kenny, Andreja Mesaric, Natalie Wilson, Vanessa Pinfold, Thomas Kabir, Daniel Freeman, Felicity Waite, Michael Larkin, Dan J. Robotham

**Affiliations:** ^1^ McPin Foundation London UK; ^2^ Department of Psychiatry University of Oxford Oxford UK; ^3^ Oxford Health NHS Foundation Trust Oxford UK; ^4^ Aston University Birmingham UK

**Keywords:** CBT, cognitive therapy, delusions, paranoia, schizophrenia, treatment

## Abstract

**Background:**

The Feeling Safe Programme is a cognitive therapy developed to improve outcomes for individuals with persecutory delusions. It is theoretically driven, modular and personalised, with differences in therapeutic style and content compared with first‐generation cognitive behavioural therapy for psychosis.

**Objectives:**

We set out to understand the participant experience of the Feeling Safe Programme.

**Design:**

A qualitative study employing interpretative phenomenological analysis.

**Methods:**

Using a peer research approach, semi‐structured face‐to‐face interviews were conducted with six people who had received the Feeling Safe Programme as part of the outcome clinical trial.

**Results:**

Participants spoke of feeling ‘unsafe’ in their daily lives before the intervention. Openness to the intervention, facilitated by identification with the programme name, and willingness to take an active role were considered important participant attributes for successful outcomes. The therapist was viewed as a professional friend who cared about the individual, which enabled trust to form and the opportunity to consider new knowledge and alternative perspectives. Doing difficult tasks gradually and repeatedly to become comfortable with them was important for change to occur. The intervention helped people to do ordinary things that others take for granted and was perceived to produce lasting changes.

**Conclusions:**

The Feeling Safe Programme was subjectively experienced very positively by interview participants, which is consistent with the results of the clinical trial. The successful interaction of the participant and therapist enabled trust to form, which meant that repeated practice of difficult tasks could lead to re‐engagement with valued everyday activities.

**Practitioner Points**

- A peer research approach can be used to explore the experience of participating in psychological therapy, providing a distinct perspective that enriches the data collected by drawing on additional lived experience knowledge.
- Consistent with the outcome findings from the randomised controlled clinical trial, participants in this qualitative study described the Feeling Safe Programme as resulting in substantial meaningful change.
- In the Feeling Safe Programme, the combination of building trust, gaining new knowledge and perspectives, and learning safety led to the positive experience of ‘a life more ordinary’.
- Participants experienced the Feeling Safe Programme as personalised. This included having choice, control, and collaboration.
- Participants described the Feeling Safe Programme as practical and active. The sessions involved entering everyday situations, both with the therapist and independently, to learn safety.

## INTRODUCTION

Persecutory delusions are when people believe others are intending to cause them harm, despite this not being the case. They are associated with negative thoughts about the self and others (Fowler et al., 2006), depression (Vorontsova et al., 2013), suicidal ideation (Freeman, Bold, et al., 2019), agoraphobic avoidance (Freeman, Taylor, et al., 2019) and hospital admission (Castle et al., 1994). Persecutory delusions are a severe form of paranoia, when beliefs are held with strong conviction. Paranoia is synonymous with fear and vulnerability, with people describing confusion, uncertainty and feeling under attack (Boyd & Gumley, 2007; Rhodes & Jakes, 2010). Paranoia can be viewed as a way for people to prepare for threats in a dangerous world. It can be an isolating experience, making it hard to see others' good intentions.

Evidence‐based treatment recommendations for treating psychotic experiences such as persecutory delusions include antipsychotic medication and CBT for psychosis (NICE, 2014). Yet meta‐analyses indicate that first‐generation CBT treatment effects for persecutory delusions are small (*d* = 0.3–0.4; Bighelli et al., 2018; Leucht et al., 2013). The Feeling Safe Programme was developed to improve the efficacy of treatment for persecutory delusions. It was based on the idea that persecutory delusions are ‘threat beliefs’, maintained by a number of psychological processes (Freeman, 2016). The maintenance factors include excessive worry, low self‐confidence, sleep disruption, intolerance of anxious affect and other internal anomalous experiences and the use of safety‐seeking or ‘defence behaviours’ (Freeman, 2016). Modular treatments targeting each factor were developed and tested in separate trials (Freeman et al., 2015). The combination of modular elements was then tested in a case series (Freeman et al., 2016). The style of therapy that has evolved from this systematic step‐by‐step approach is akin to interval training: bursts of activity followed by periods of reflection and integration (Freeman & Waite, 2017). Therapeutic time is dedicated to the implementation of strategies in day‐to‐day life, which includes additional contact (e.g. telephone calls) between the weekly sessions.

A Randomised Controlled Trial (RCT) with 130 patients who experienced persistent persecutory delusions in the context of non‐affective psychosis has compared the Feeling Safe Programme to a befriending intervention delivered by the same therapists (Freeman et al., 2021). The Feeling Safe Programme produced large reduction in persistent persecutory delusions above those of befriending. This trial showed that the programme has produced the largest treatment effects yet reported for persistent delusions, and the size of clinical benefits was closer to those found for targeted cognitive therapy approaches for people with anxiety disorders.

But what was participants' subjective experience of the programme? To answer this, we conducted a qualitative study with a subgroup of participants who had received the Feeling Safe therapy. Our study used ‘peer research’ methods; key members of the study team had experienced persecutory delusions, like the participants. The aim of this study is to understand participants' views and subjective experiences of the intervention to inform the future development of the programme. This study was conducted independently of the therapists and trial team to increase the likelihood that participants would give frank accounts of their experiences.

## METHODS

We conducted a qualitative semi‐structured interview study of participant experiences, using peer research methods (Yang & Dibb, 2020). Participants were recruited to the study having received the Feeling Safe intervention in the randomised controlled trial. We took a phenomenological and idiograhic approach, combining Interpretative Phenomenological Analysis (IPA) and Template Analysis (TA; Smith et al., 2021). The study had received ethical approval from an NHS Research Ethics Committee (South Central ‐ Oxford B Research Ethics Committee: ref 15/SC/0508). Informed consent, in addition to the main trial consent process, was obtained from all participants for the Feeling Safe experience study.

### The Feeling Safe Programme

The programme was provided individually in approximately 20 sessions over six months. The programme is modular, personalised and tailored to people's preferences. Following the assessment, participants were presented with relevant treatment options and choose what they would like to do, and the order in which to do them. Participants completed a median average of three modules. The most frequently completed modules were (in order): feeling safe enough (dropping of safety‐seeking behaviours to reduce threat beliefs and develop new memories of safety), improving self‐confidence, reducing worry, coping with voices, and improving sleep.

The programme was framed to participants as aiming to help them feel safer, happier and to enable them to do more of what they want to be doing (Freeman & Waite, 2017). Modules were delivered one at a time. Participants were encouraged to use strategies they had practised with the therapist between sessions, supported by phone calls and/or texts.

### Lived experience involvement

A patient advisory group of four members advised on the study (and on the trial itself). Data collection and analysis used a peer research approach (Yang & Dibb, 2020). Interviews were conducted by people with similar life experiences to the participants, and that this was disclosed as appropriate. A peer research approach is ‘well suited to understanding lived experiences’ (Woodall et al., 2019). It allows for nuanced data collection and analysis. For example, a shared understanding of language, and empathy based on similar experiences can enhance rapport, reducing the boundaries between researcher and participant (Terry & Cardwell, 2016) and levelling power (Harding et al., 2010). This can result in the collection of more open, honest and detailed data (Harding et al., 2010; Videmšek, 2017). During analysis, peers bring different perspectives and insights (Gillard et al., 2010; Rose, 2008; Sweeney et al., 2013).

Data were collected and analysed by the peer research team. This included two researchers who had similar life experiences to the participants (one recruited from the patient advisory group). They worked alongside two other researchers who had an experience with mental health issues, though unrelated to the experiences of the participants. During analysis, researchers kept reflective logs documenting how they drew on lived experiences. After initial analysis, some interpretations were shared with the advisory group, where members' comments deepened the interpretations.

### Sampling and recruitment

An idiographic and phenomenological approach involves a commitment to in‐depth data and case‐level analysis. We therefore sought a relatively small sample size. Consecutive trial participants from a six‐month cohort were invited to participate. The study reports on the analysis of six participants who had received the programme.

### Participant characteristics

Six participants were interviewed, four men and two women. The average age was 41 (range 22–62 years). Four people identified as White, one as Indian and one as Black‐African. Five were single, one was married or in a civil partnership. One was self‐employed at the time of the interview, and all other participants were unemployed. Three people lived alone, two with parents and one with a relative/spouse.

Participants were asked about how they felt before the intervention. Most spoke from the perspective of feeling ‘unsafe’. Three indicated that they felt unsafe in their home or neighbourhood. All spoke of experiencing paranoia, hearing voices, having trouble sleeping and of anxiety about being in public places. Two spoke of losing confidence, and one about experiencing depression and suicidal thoughts.

### Data collection

Semi‐structured interviews took place between October 2018 and April 2019 and lasted around 40 minutes (range 19–77 min). The interview schedule was developed in collaboration with the patient advisory group. It focused on the participants' situation prior to intervention, experiences of the intervention and perceived subsequent effects. Interviews occurred face‐to‐face, in safe and private locations where participants felt comfortable. Interviews were audio recorded and transcribed verbatim.

### Data analysis

Interpretative Phenomenological Analysis (IPA) focuses on understanding an individual's lived experience and how they make sense of it (Smith et al., 2021). It complements peer research because it acknowledges the role of the researcher (Bond et al., 2021). We looked at two cases in‐depth using IPA, exploring the phenomenological experience of individuals in relation to their experiences of their symptoms and their journey through the intervention. A Template Analysis (TA) approach was then used to explore all six interviews. TA is a flexible approach, which involves developing and applying a coding ‘template’. The result is a summary of themes in a hierarchical structure, in which broad themes encompass successively more specific ones. TA can be used alongside IPA (Brooks et al., 2015; King, 2004). The two methods ensured depth and breadth.

The combined analytic process involved two researchers reading the IPA transcripts and making line‐by‐line descriptive, linguistic and conceptual notes on each interviewee's claims, concerns and understandings (as described by Larkin & Thompson, 2011). They mapped interviewees’ phenomenological experiences by identifying what matters to them (their ‘objects of concern’) and the meanings they assigned, then identified common themes from these maps. These themes were used to create a ‘template’, an a priori list of codes analysed via TA (informed by the approach of King, 2004). Codes were assigned systematically from the template using NVivo software. The template was updated following this process. All transcripts were read again, with the coding and template revised accordingly. Themes were written up, supported by illustrative quotations from participants. The wider research team and the patient advisory group provided feedback.

## RESULTS

Findings are presented in two sections. The first presents the cross‐case analysis of participants' experiences of the intervention. The second presents detailed case studies of the two participants whose interviews were analysed using IPA. All names are pseudonyms, and details such as place names have been omitted to ensure anonymity.

### Across case analysis: Exploring experiences of feeling safe

Quotes from interviews were chosen to illustrate each theme within the text below. Some other quotes are presented in Table 1 to demonstrate how the theme manifests for other participants.

**TABLE 1:** Illustrative quotes from participant interviews

Theme	Participant	Extract
Engaging with everyday life	Helen	I was able to go out that bit more and try and lead as much of ordinary life as possible
	Colin	2 I started doing volunteer work stuff like that, so I've been doing that and going to town three or four times a week, going to the shops every day
	Ed	3 I'm much more outgoing than I was now. I'm, sort of, on the up in a way, I've got a job and I'm applying for lab jobs and things like that and all those things make you feel good about yourself
Openness, engagement and personal responsibility	Rajiv	4 So I thought, ‘At least I'll get a companion to actually listen to me. What harm can it attract?’ So I enrolled in the programme
	Helen	5 So, other things had not worked for me so I thought that surely something has got to work…Part of me thought, ‘I've got to try it’. And part of me was very sceptical. You know, ‘How can talking about it make it better?’
	Rajiv	6 I knew that the road is not easy but then to actually get well I have to go through this
	Helen	7 I thought to myself that if I'm going to do this, I've got to go for it, I cannot hang back so I sort of psyched myself up to it
Professional friend	Ed	8 I got to know (therapist) a bit better and felt much more comfortable around her and comfortable to say what was on my mind and I guess I gained a bit more faith in the study and what they were trying to do really
	Helen	9 Because I was lonely, that was a lot of my problem. So, I'd come to see them as friends and it would have been difficult not seeing them again because that's me on my own again, so, whether I would have slipped back a bit, I do not know?
	Rajiv	10 She said, ‘There's always a number you can call me. You can call [therapists]. You can call any one of us, how it's going. Even if it's a good thing you want to tell, you can tell us. You do not need to always discuss your problems, you can also discuss about these things. We are always there’
	Adriana	11 I felt very comfortable and like there was a friendship between us. And it was kind of like if I had not seen her for a while, then it feels like a long time
New knowledge and alternative perspectives	Adriana	12 The [therapists] helped me to think that, you know, that it's not about me: what they are doing, whether they are smoking or spraying—it's not about me. It's just because of their own experiences because that's what they do. It's not connected to people in my country, like political reasons, because that's what I was thinking
	Keith	13 The information has not been there for me and so doing the Feeling Safe project, I did learn quite a lot from that and learnt new things about my illness which I did not know
	Rajiv	14 …putting that balance in place [of when fear is justified and when it is not] also was a good thing from the Feeling Safe Programme. From my side, I was amplifying everything. So I think my brain was taking a small thing, ‘It's morning, if I go outside and I'm trying to step into the train, what if I step in the gap?’ all those small things
The right pace	Helen	15 …starting off was quite, sort of, low level, gently getting into it and then, towards the middle and the end, it was really very full‐on sessions, really
	Keith	16 The last two months we went out on the bus and went into cafes. I had a bad habit of sitting facing the main doors to watch people come and go. Then over time, I was able to turn my back to the door and let people come in from behind. We went on the bus several times to (town) to break the habit of me getting paranoid about people… It was just breaking the cycle really 17 We did all the talking and going through the paperwork and all that. We started off going to cafes to start off with, just to see how it went. I found it uneasy the first time doing it but I think a few weeks into it was getting easier as time went by, learning new techniques to deal with the situation
	Colin	18 It just got a lot easier. Once I started doing it, just keep doing it. It's a lot easier over time
Flexibility and fitting	Adriana	19 The [therapists] did it according to my personality. It fitted my personality, I think. Yes. So, other than the challenges of doing the work, it kind of fitted with me
	Keith	20 I think [the intervention] covered every angle. With my personal issues, I cannot see it being any better for me really
	Ed	21 …probably the most helpful I found it was when it came down to the revision and (therapist) taught me different ways to revise and different ways to memorise information. So yes, it was definitely helpful
	Rajiv	22 I always had an inclination towards exercising and waking up in the morning to do things before these things [the voices] came to me. So somewhere it was there…I knew how to achieve it. But then they gave me positiveness saying, ‘You need to go towards that side of it’

#### Engaging with everyday life

After the intervention, all participants experienced positive changes in issues that bothered them, which they attributed to the programme. People talked about anxiety and paranoia reducing, sleep and confidence improving, being more able to cope with voices and relinquishing safety‐seeking behaviours. Two people identified as no longer having psychosis.

As well to changes in mental health problems, the intervention helped people do ‘ordinary’ things, such as walking the dog, eating out or maintaining family relationships. For some, there was a sense that life had expanded, geographically and/or personally. People now had the confidence to travel further afield and use public transport, and there was a sense of personal growth in terms of volunteering, applying for jobs and reentering the social world. One person referred to a shift in the way he thought about himself. Both these dimensions are illustrated by Ed in his ‘I'm on the up’ extract (Tables 1 and 2) and here:

So, I think everything has improved, to be honest. My outlook on life, the way that I carry myself, my mental health, in general, and I think the Feeling Safe study, definitely had a strong role in that transformation. Ed

**TABLE 2:** Additional features of the therapy

Feature of therapy	Participant experiences	Participant quotes
Setting	Location Flexibility about where the sessions took place—including home visits and going out into challenging situations together—was appreciated	They had a whole load of rooms there [in the hospital], half a dozen, I think, that were just chairs, where you could just sit and talk. So, it was quite good there, it was quite relaxing there but I definitely preferred going out somewhere different
		She would come to my house, which made it really easy and we would just sit in my dining room and talk about that
		I think her coming here was very helpful because as I say, my energy is low and by the time finish shower, getting ready and travelling to the hospital, it would have taken so much energy that I have not got…So, her coming here was really, really helpful because all I have to do is shower [laughs]. Yes
	Length of session An hour session was ‘about right’, ‘fine’, ‘just right’, with sessions sometimes lasting longer if they were out and about. People who commented on the amount and intensity of the contact referred to it as being ‘just right’, ‘about right’ or ‘plenty’	There was a lot of flexibility in it. You had enough time to do your session and complete it. You did not have to end it, stop halfway and then continue following on the following week because you can forget sometimes what you have learnt in the first session
		They were quite intense but not too intense, they were good…I left the sessions feeling like I'd taken on what she was trying to say and not too much was put upon me, yes that's what I would say
	Contact between sessions Therapists contacted participants in between their sessions. The aim of this was to check how they had found the previous session, how the goals the participant had set for that week were going and if there were any difficulties. One person said that text messages may have been easier initially, when she was getting to know the therapists	It was just right. I think if you had too much contact, I do not think it would have really worked
		She was checking how I was and also if I'm doing the things that I'm supposed to do like going for a walk or what time I've been going to sleep
Style	Collaborative decision making Most participants agreed that their views about treatment were listened to and they felt involved in decisions, such as which modules to work on and which places to visit together	I think it was nice because you do not get bored with one choice or if you find something challenging, at least then you move on to another topic. So, that was good
		I think we selected [the module] together and we talked about it but I think there were two or three and I cannot remember what they were, now. So, there was definitely no, sort of, ‘You're going to do this whether you like it or not’. It was just that we talked about it
	Active participation Some participants described actively learning new approaches and techniques during the therapy	You were learning different techniques to deal with things
	Practice between sessions Participants recalled being asked to do tasks in between sessions, including things that previously they would have avoided Some people found this hard or struggled with the motivation to do it independently	I was set small goals. We'd talk about an issue and then we'd find out the problems I had in those issues. I'd go away and try to bring those issues into action. So what I learnt, I was able to practice on other people if people would cause me problems. *Was that helpful to have tasks to complete in a way between sessions?* Yes, because it was a chance to do it. If I had any issues, in the next session we'd talk through it and then we'd find ideas or ways to deal with it
		When the therapist was here, I was alright because it's kind of like you are talking and you are having a laugh and you are doing… and then I enjoy it. But when I was doing it on my own, I found it difficult. I find it a chore really
		I did with the walking down the road bit but I found the voices hard, very hard to do that
Materials	Therapy booklets Participants were provided with written materials during therapy. One person referred to going back to the materials when he needs to, treating them as a continuous learning resource. Another recalled that the handouts gave ideas of things to do to keep you busy and someone else that having things written down helped reinforce the things that she and the therapist spoke about	Yes. I've still got the paperwork in a folder. If I've got any issues, I can go back to the paperwork and look up and find the solution to my problem. I'm still learning today how to deal with things. As I say, I go back to the module to look at things, just to read up on a few issues

Everyone said that the positive changes had lasted since the therapy stopped. This was attributed to better sleep, a more positive outlook, lifestyle changes that meant they became more social and physically healthier and being better able to understand symptoms.

#### Openness, engagement, personal responsibility

From participant accounts, it was evident that openness to the intervention was important to the therapeutic interaction. Participants felt that Feeling Safe was suitable because they identified with its name, because it gave them someone to talk to and because they felt that eventually something had to work for them. Some were sceptical, as they perceived a mismatch between the gravity of their difficulties and the seemingly simplistic approach of talking therapy. Scepticism could exist alongside the hope that it would work.

I was quite intrigued to see how it worked and see if I'd benefit from it. Keith

Participants felt that taking an active role in the intervention increases its potential to have a positive impact. Four participants acknowledged the need to challenge themselves, one also talked about the importance of self‐belief when doing so. Although participants sometimes found activities difficult, they accepted that challenging themselves was necessary. This caused internal dilemmas: One person spoke of tension between the need to be honest with the therapist about his fears, alongside a reluctance due to knowing he would have to face them. Another spoke of ‘forcing’ himself to leave the house. One mentioned returning to the Feeling Safe booklets after the intervention had ended, with another considering how he could maintain changes while still in therapy.

I asked her to actually narrate whatever we did, talk about it and then give some instructions which I should be doing, that kind of thing might happen…Then I actually played that more than three or four times after that programme at certain places where the doubts start coming into your mind. Rajiv

The concept of personal responsibility is featured in four of six participant accounts, for example:

I thought to myself that if I'm going to do this, I've got to go for it, I can't hang back so I sort of psyched myself up to it. Helen

In one of the remaining accounts, the participant described the need and challenge of active change. This person had experienced beneficial changes from the programme, such as improved sleep and reconnecting with family, and acknowledged that they were still working towards their goals:

What I found is that I am not yet there, if that makes sense. I'm still struggling with doing everything. Adriana

#### Therapist is a professional friend

For all participants, the relationship with the therapist was central to the intervention. People described the therapist(s) as ‘friendly’, ‘helpful’, with some commenting on the ‘comfortable’ atmosphere they established. Participants who were initially sceptical said that developing this relationship was key to gaining trust.

It was just relaxed and, not like having a friend, but like that. They made it as relaxed as possible, really, so, talking about the dog and things, to begin with. So, I don't know, discussing other things other than what I was going to be doing in the study. Helen

Some participants, including two who struggled with loneliness, experienced the therapist as being ‘like a friend’. The therapist asked about participants' lives beyond their problems or symptoms. Two participants mentioned they were encouraged to phone the therapist after sessions had finished, if there was a problem, or if they wanted to talk. This reinforced the sense of trust and that the therapist cared.

Therapists providing their phone numbers may imply that the therapist trusted them to use them appropriately. No one mentioned calling the therapist. Having the option to call them may have been reassurance enough. People enjoyed the therapist's company and looked forward to sharing experiences and getting their perspective. Activities were more enjoyable in their presence. One person mentioned missing the therapist if there was a longer than usual gap between sessions. Even for the person who did not talk explicitly about friendship, there was a sense of the therapist genuinely caring for them.

I think they care about what they're doing, and they just want to help their patients. Ed

#### Therapist knowledge and perspective

The therapist brought new knowledge and alternative perspectives. All but one interviewee referred to the intervention enabling them to understand their fears or difficulties differently. Two talked about the impact. One implied he had been unwell for some time before starting sessions, so this enabled him to understand himself and his life more: ‘why your illness was causing you to be the way I was’.

This theme arose less often and with less emphasis than the ‘Therapist as a professional friend’. Based on discussions with the patient advisory group and one of the peer researchers, who spoke of the importance of receiving this type of information at the right time and context, we suggest this may be because participants were at different stages of their recovery journeys, and able to engage with this information to different degrees. Since participants were given booklets to keep, people could return to information when circumstances were more conducive, something that members of the group, and the peer researcher, had done.

Patient advisory group members recalled therapeutic interactions that led to ‘epiphanies’ and ‘revelations’ enabling new mindsets. One participant described seeing the graph of anxiety reducing as he practised new approaches to situations.

One of the things that I've good memory of, is this graph that she showed me, that for every time you do something, you become less and less anxious… Even though it seems obvious, to me at the time, where I was, with my mental health, it just resonated with me, that yes that you could be feeling better after a week, if you follow that and challenge yourself. Ed

The patient advisory group spoke about how empowering this could be, something that Rajiv felt (see his case study, below), and how it could reduce self‐blame and increase self‐compassion.

Given the potential power of being provided with timely new knowledge, this theme feels like an important and essential element of the intervention despite not being a salient aspect of therapy for everyone.

#### The right pace

All interviewees valued the gradual nature of the approach. The initial period spent at participants' homes working through the psychological modules, which participants experienced as talking about their problems, was important because it enabled rapport and trust. This was a necessary foundation for the next step, visiting previously feared places or scenarios, as participants needed to trust the therapist before putting themselves into feared situations. Having the therapist alongside them was important.

Doing difficult tasks repeatedly was also part of becoming comfortable with the task, particularly for people who were trying to change safety‐seeking, or defence, behaviours, such as sitting in a particular seat in a cafe. At some point, participants were encouraged to visit places or do activities on their own, in between therapy sessions. Again, this required the participant to trust the therapist's judgement that they were ready to take this step. Some found this acceptable, while others persevered before it became easier. This gradual, repetitive approach in conjunction with the trust in the therapist built safety into the intervention.

I think I got enough information because if they had told me more, I probably would have been anxious. Do you see what I mean? If they had told me, ‘Oh, you're going to go for a walk on your own’ I'd probably be more anxious expecting that. Adriana

#### Flexibility and fitting

Some participants spoke of the intervention aligning with their personality, or with their difficulties (‘it kind of fitted with me’). This does not stem solely from having a choice about how the therapy was conducted, because participants struggled to recall details about choosing the psychological modules. Take Ed for example. He talked less about the psychological modules, referring to ‘techniques’ but only elaborating on ones he did not find helpful. As expected, he and the therapist worked on visiting places he perceived as unsafe, but the therapist also helped with life skills, such as driving and revision techniques (which he appreciated).

One participant talked about doing role plays to prevent people from coercing him into doing things for them. Another described Feeling Safe as a multifaceted intervention, returning to positive lifestyle changes he was encouraged to make (diet, exercise and reducing alcohol consumption). This was something he had a prior interest in doing, before becoming unwell.

It was as a result of therapy, definitely, because when you are actually going towards the positive side and you're talking about all these positive experiences… one thing I knew in the back of my mind, if I'm not good physically then I can't get it. I can't take it mentally, so I need to be physically active, get that mental thing down. Rajiv

The variation in the accounts suggests that flexible routes to progress matter to participants. The patient advisory group and the peer researcher identified with this. Participants felt that the intervention was a perfect fit for them, which may contribute to motivation and engagement. It may also have been another way of conveying that it was safe to take risks within the confines of the intervention. If the therapy was tailored, they could trust the therapist to only ask them what they were capable of, not force them on a rigid treatment path.

#### Sufficient time for change to occur

At 20 sessions over 6 months, the duration of the intervention is consistent with the minimum of 16 sessions recommended by the NHS for first‐generation CBT for psychosis [NICE, 2014]. When prompted, participants said that the frequency and duration of the therapy were ‘just right’ or implied that this was the shortest time required for change to occur, but no one spontaneously mentioned the duration of therapy. This is a background phenomenon required for the provision of gradual, tailored approaches valued by participants.

### Exploring two participant accounts

We present the case studies of two participants who described their experiences in the most detail. These were analysed using IPA. These accounts demonstrate how two people took an active approach to the therapy and how this connects to other elements of the intervention. This theme is the focus because it is the most important individual element relating to the participants and underpins their level of engagement with the intervention.

#### Ed: The importance of challenging yourself

Ed was diagnosed with psychosis and referred to an Early Intervention in Psychosis service while at university. As well to hearing voices, he experienced paranoia and felt that certain places were unsafe and inhabited by hostile people. He was initially sceptical that the intervention would work but was open to engaging with it. It helped that the therapy moved at a pace that suited him, beginning with talking to the therapist then going outside together (see ‘The right pace’ above).

It, sort of, grew over time, initially it was meeting with (therapist) and she'd give me different techniques… she would come to my house, which made it really easy and we would just sit in my dining room and talk about that….

But we did a lot of work, because at the beginning, I was so anxious about leaving the house that we started just by going down my road and into the woods and walking about in the woods.

The approach succeeded:

…as the year progressed, I grew more outgoing, got a job and I don't know, moved on a bit, I guess and became more able to go outside and I guess that was the exposure element.

The concept that Ed refers to as ‘exposure’ involves going into feared situations to build the safety belief (e.g. I am safe enough to do the things I want, to go to a café or walk the dog). For Ed, discovering that anxiety reduced meant that he was not under threat, although he sometimes felt anxious. The therapist gradually building the intensity of this practice meant that eventually, Ed was able to visit a city he felt was unsafe. His use of ‘adventurous’ below suggests that he relished the challenge, at least in retrospect.

Yes, that's what I'd say, a balance of stress and exposure … and as time goes on, you become more adventurous and able to deal with more and that's when I started to go places like (city), (city), (distant city).

The therapist also helped Ed with life skills, such as driving and revision techniques (see ‘Flexibility and fitting’ above). Passing exams and a driving test are milestone events and can be seen as part of a transition to adulthood, so it is unsurprising that this support was so meaningful to Ed, who experienced his first episode of psychosis while at university.

Something that varied in the account is the extent to which Ed presents as an active participant in therapy processes (see ‘Openness, engagement and personal responsibility’, above). When recalling how he got involved in the study, he gives the impression that he sees himself in a passive role, grateful for the expertise of the healthcare professionals and their ability to fix him. There is a subtle shift when he talks about the need to practice (in his words, the ‘exposure’). Ed acknowledges his previous passivity and talks about having enough time for behaviour change to occur. The culmination of this is an explicit acceptance of the inevitable hard work:

I guess the Feeling Safe study taught me that the things that you fear the most, you have to do and to get over them and that's just the way it is

#### Rajiv: Changing thought processes gives a sense of control

Rajiv began hearing malevolent voices 18 months before the interview. At work, he was careful to hide his experiences. He seemed like someone who enjoyed being around people but when his difficulties began this was replaced with anxieties about being judged and fears of crowded places. A strong theme throughout is his attempt to make sense of what is happening.

It was his friend's wife who first suggested that the voices might be coming from within, which led to a diagnosis and medication. The day after, ‘… I got rid of my sense that the things are outside, they are inside me’. Despite some initial scepticism, Rajiv enrolled in Feeling Safe and embraced the opportunity to talk about what was happening with trusted people. Speaking with the therapists, reading their books and observing people going about their lives helped Rajiv accept the internal origin of the voices (see ‘Therapist knowledge and new perspectives’, above). This enabled him to better cope as he could frame the voices as annoying rather than frightening. The voices had become something he could make sense of or choose to ignore.

Even if it's a real thing or it's an unreal thing, now I try to ignore what I should ignore. So I'm just developing that kind of learning of things.

Understanding his own role enables him to feel more in control of his fears and understand that he can consciously work towards changing the contributing thought patterns rather than rely on other coping strategies: ‘Now I know that I don't need a drink to counter this. I need a thought process to counter a thought process’. This had an empowering effect and Rajiv began to look for ways to challenge himself (see ‘Openness, engagement and personal responsibility’, above).

It has started feeling like a win. So I was looking for smaller things which I am trying to overcome because you need to believe that you can overcome this.

At the time of the interview, Rajiv identified as cured and was no longer taking medication. He was sleeping better and he had found a new way to think about his voices. He cited both Feeling Safe, and the medication he had previously taken, as reasons for his improvement. The non‐judgemental, ‘safe’ human interactions were also an essential part of the way back to ordinary life (see ‘Therapist as professional friend’, above).

I never knew the power of the real things and the talks when you actually share things and talk to people, not yourself, when you talk to someone else …[Feeling Safe] gives you a companion to share something. That's where it begins because most of the people with these kinds of psychotic things, the first thing they do is they don't know who to share with.

## DISCUSSION

Participants' experiences of the intervention were positive, and the intervention was perceived as enabling meaningful changes. Participants perceived Feeling Safe as practical, solution‐focused and tailored to their needs and interests. They described the intervention in terms of their experiences with it, and the relationships involved; the therapist visited the participant's home and talked about their problems, and they went outside and practised challenging tasks, first together and then alone. As one member of our patient advisory group said, this is a ‘more natural’ form of therapy than one that is confined to a therapy room. Participants rarely mentioned the individual modules of the intervention, but some spoke about learning new information and being given alternative ways to appraise troubling thoughts and feelings.

Therapeutic trust was fundamental, allowing participants to ‘feel safe’ and to take risks in the therapy. This is important because participants did challenging, feared or uncomfortable tasks, gradually but repeatedly. The presence of the therapist, who the participants perceived as a ‘professional friend’, supports participants to ‘feel safe’ in this situation. Those participants felt able to be themselves and were seen as individuals by the therapist likely helped as the participant would have felt that the therapist understood them and knew what they were capable of. One member of our advisory group remarked that having the therapist visit people's homes and then accompany them with everyday tasks may contribute to this feeling of being ‘known’ and able to trust what was asked of them. It may have also humanised the therapist, rather than them just being seen as a health professional. The importance of a therapist who is emotionally invested and interested is consistent with the findings of previous reviews of qualitative studies of CBTp (Pipkin et al., 2021; Wood et al., 2015). In a meta‐ethnographic review, participants described the use of collaboration and shared agency as promoting a sense of self‐efficacy and levelling power in the therapeutic relationship (Pipkin et al., 2021). In Feeling Safe, joint decisions were made about which tasks to do, and the order in which to do them, and there was flexibility within the intervention to focus on what mattered to the individual. This meant that participants' overall experience of the intervention was that it was responsive to their needs and that they were in control, again adding to a feeling of safety.

Previous qualitative studies of the experience of CBTp have found that increased understanding of coping mechanisms and the provision of alternative perspectives along with improved social functioning (Berry & Hayward, 2011; Wood et al., 2015) are valued therapeutic outcomes for patients (Holding et al., 2016). Yet long‐term behavioural change can be difficult to achieve (Kwasnicka et al., 2016). In Feeling Safe, behavioural change occurs through practice in real‐life situations to help the participant learn (or relearn) a sense of safety and enable them to lead a more ‘ordinary’ life. This occurred in the context of openness to the approach, taking on the responsibility to try challenging tasks, provision of new information and a trusting therapeutic relationship. The architecture behind the modular intervention was often invisible to participants, but the learning made from the whole experience was prominent.

Our findings are commensurate with previous user‐led studies, which show that people with lived experience tend to emphasise the emotional and relational aspects of care (Gilburt et al., 2008). The Feeling Safe intervention was resource intensive in terms of time and therapist input. The time‐intensive aspects of the intervention included the willingness to work on practical tasks that the participant was interested in, the weekly sessions and interim phone calls, and participants being given permission to call them. These aspects were important because they enabled the building and maintaining of trusting relationships, but they need to be managed according to therapeutic boundaries. Since two Feeling Safe participants were worried about the intervention coming to an end, it is important for therapists to handle endings well, and frame the relationship as a professional interaction. The nature of the intervention lends itself to therapists who are experienced in handling complex, interpersonal situations in therapies tailored to individual participants.

The analysis described above focuses on the elements of the intervention that are important to the participants. It is based on what matters to them and the meanings they assign to these things. During the interviews, participants were asked about other aspects of the Feeling Safe therapy, which may be useful for people carrying out Feeling Safe in future. These are summarised in Table 2.

This study was carried out over a long duration and two pairs of researchers conducted the study. The data were collected by one pair and analysed by a second pair. Both pairs combined peer experience of persecutory delusions with peer experience of unrelated poor mental health. Over the duration of our write up, we lost members of the patient advisory group, and only two original members were present for the synthesis sessions. New members were invited from a similar group. We adapted our peer methods but lacked field notes from interviews to contextualise transcripts such as physical cues to help re‐contextualise a setting or moment in the interview. All researchers commented on the final draft of the paper, providing reassurance that it honoured the data with integrity and care. This was corroborated by the perspectives of the patient advisory throughout the analysis and writing process.

The participant size was limited to six people. These participants had completed all therapy sessions and were positive about the intervention. We did not hear from people who may have been less positive. However, our in‐depth, subjective approach to analysis provides an understanding of how the therapy impacted on people who engaged with it. Findings are limited by our own interpretation of the data. Other interpretations could have been made by different researchers, but analyses were strengthened by lived experience expertise, which was embedded throughout the study, and validated through our patient advisory group. Overall, the study provides an important subjective account of a new intervention for persecutory delusions that have shown large clinical effects on established quantitative outcome measures.

## AUTHOR CONTRIBUTIONS

The study was conceptualised by DF, FW, and TK. TK facilitated the Patient Advisory Group. Data collection was completed by AM and NW. Data analysis was completed by JB, AK,VP, and DR with supervisory input from ML and FW. The manuscript was prepared by JB, FW, DR, and DF. All authors contributed to and approved the final manuscript.

## CONFLICT OF INTEREST

There are no conflicts of interest.

## Data Availability

The data that support the findings of this study are not publicly available: Due to privacy, it would not be ethically appropriate to share the whole data set. However, selected quotes to support claims made in the paper are available upon request to the first author.
